# Efficient Graphical Algorithm of Sensor Distribution and Air Volume Reconstruction for a Smart Mine Ventilation Network

**DOI:** 10.3390/s22062096

**Published:** 2022-03-08

**Authors:** Yujiao Liu, Zeyi Liu, Ke Gao, Yuhan Huang, Chengyao Zhu

**Affiliations:** 1 College of Safety Science and Engineering, Liaoning Technical University, Huludao 125105, China; liuyujiao@lntu.edu.cn (Y.L.); liuzeyijy@163.com (Z.L.); zcy1339645348@163.com (C.Z.); 2 Key Laboratory of Mine Thermo-Motive Disaster and Prevention, Ministry of Education, Huludao 125105, China; 3 Centre for Green Technology, School of Civil and Environmental Engineering, University of Technology Sydney, Sydney, NSW 2007, Australia; yuhan.huang@uts.edu.au

**Keywords:** mine ventilation network, wind speed sensors distribution, air volume reconstruction, independent cut set

## Abstract

The accurate and reliable monitoring of ventilation parameters is key to intelligent ventilation systems. In order to realize the visualization of airflow, it is essential to solve the airflow reconstruction problem using few sensors. In this study, a new concept called independent cut set that depends on the structure of the underlying graph is presented to determine the minimum number and location of sensors. We evaluated its effectiveness in a coal mine owned by Jinmei Corporation Limited (Jinmei Co., Ltd., Shanghai, China). Our results indicated that fewer than 30% of tunnels needed to have wind speed sensors set up to reconstruct the well-posed airflow of all the tunnels (>200 in some mines). The results showed that the algorithm was feasible. The reconstructed air volume of the ventilation network using this algorithm was the same as the actual air volume. The algorithm provides theoretical support for flow reconstruction.

## 1. Introduction

Coal is a primary energy source and plays a key role in economic development in many countries. In China, coal accounts for 40–45% of total carbon emissions [1]. Meanwhile, due to the unique working environment in coal mines, the requirements for safe operations are very high. The working environment affects the safety of those working in coal mines. Mining accidents could cause significant loss of life, as well as economic losses to a country. In 2020 alone, 573 Chinese miners died in 434 mining accidents such as leaks of poisonous gases, explosions of natural gases, and the collapsing of mine stopes, especially from underground coal mining [2]. An efficient and reliable ventilation system is essential for safe and efficient production of coal [3].

**Figure 1:**
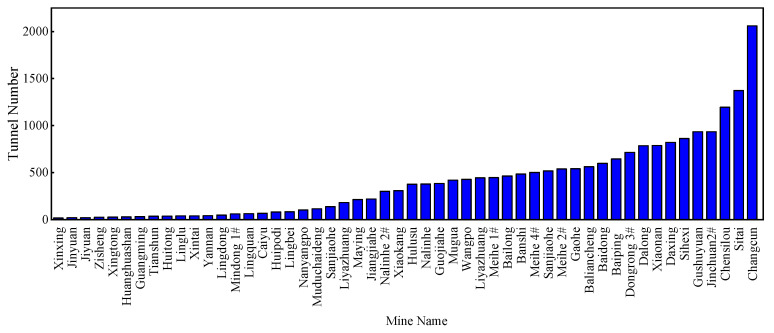
The number of tunnels in mines in China.

Intelligent ventilation is a development trend for coal mines and other types of mines. The Chinese National Development and Reform Commission and eight other ministries jointly issued *Guiding Opinions on Accelerating the Intelligent Development of Coal Mines* to improve the intelligence level of coal mines. A set of systems, including development design, geological guarantee, mining, transportation, and ventilation, are needed to achieve the goal of intelligent decision-making and automation systems operating collaboratively by 2025 [4,5]. Intelligent coal mining is to be fully realized by 2035 [6]. The reliable operation of mine ventilation systems is the basis for safe production in intelligent coal and noncoal mines. Intelligent mine ventilation is a new ventilation system that can be adjusted automatically on demand, which is the development trend of mine ventilation technology in China [7]. Proper monitoring of airflows for all tunnels plays a key role in the development of intelligent mine ventilation systems [8,9]. Airflow monitoring depends on sensors. Hu [10] studied the influence of wall roughness on wind speed distribution to improve the accuracy of monitoring. However, mine ventilation is a very complex network system involving hundreds or even thousands of tunnels. As shown in Figure 1, the Changcun coal mine in Shanxi province, China has 2061 tunnels. It may be impossible to install wind speed sensors in every tunnel due to the high installation and maintenance costs. Air monitoring was highlighted in the assessment of mine ventilation systems and air pollution to improve health and safety for miners [11]. Therefore, it is of great importance to economically determine the minimum number of wind speed sensors that can sample sufficient information to accurately reconstruct the whole network. Airflow reconstruction consists of well-posed reconstruction and underdetermined reconstruction. The so-called well-posed reconstruction is to reconstruct the airflows of the whole network with the minimum number of sensors when the number of sensors is sufficient. The underdetermined reconstruction is realized when the number of sensors is insufficient. The independent cut set algorithm is a well-posed airflow reconstruction algorithm.

A fully visual environment of ventilation parameters is very important to realize intelligent ventilation of mines. In order to visualize the air volume, an air volume reconstruction algorithm is proposed in this paper. In past studies, optimization and reconstruction technologies of sensor networks were widely used in flow monitoring [12], for the development of intelligent systems [13], and for leak location diagnosis for water [14,15,16], oil [17,18], and gas [19,20,21] pipeline networks. Singh et al. [22] proposed a method to identify ideal monitoring sites for water quality. Huang et al. [23] established a PSO-LSTM model to realize the identification of sources with abnormal radon exhalation rates. Castillo et al. [24] proposed a method of flow reconstruction by constructing a matrix of constraints to give bounds on the number of sensors required. In this method, the construction of a constraint matrix depends on all possible paths of the network. For complex networks, the measurement process in this method will also be very complex. The method was optimized to reduce the impact of network complexity [25]. However, the construction of a constraint matrix depends on the network path; although it has been optimized, the construction process is still very complex. Rinaudo et al. [26] proposed a minimum number of sensors arrangement method to determine the temperature. Although this method reduces the cost of monitoring, the measurement process is too complex. Balaji et al. [27] proposed a method to maximize sensor lifetime by activating sensor covers one at a time to monitor the whole network. This method mainly depends on the effective usage of available resources. Li et al. [28] put forward a novel method based on deep learning techniques and transfer learning to deal with large-scale missing data problems. Ng [29] proposed a method to calculate the number of sensors without depending on the path. He [30] developed an efficient algorithm to determine the smallest subset of links in a traffic network for sensor installation. Muduli et al. [31] proposed a novel wireless sensor network deployment scheme for environmental monitoring in longwall coal mines, which provided the best area coverage and was very cost-effective. Automated ventilation system adjustment software has been developed by monitoring the air volume of the minimum remainder branch in the network, and all air volumes of the branches in the network can be obtained by inverse calculation of associated branches [32]. Song et al. [33] proposed a method for determining the changing trend of gas concentration in the goaf. Lyu et al. [34] proposed a gas concentration prediction method based on the ARMA model, the CHAOS model, and the encoder–decoder model (single-sensor and multisensor). Foorginezhad et al. [35] proposed advanced sensing systems utilized for relevant monitoring and recommendations for improving sensing accuracy.

Mine ventilation networks are similar to traffic networks on topology. Mine ventilation networks are high-order nonlinear systems [36]. In the literature, different approaches have been used for determining the minimum number of sensors needed to identify the location of a ventilation system’s faults [37], such as the coverage of node flowrate method [38], the least full-coverage distribution method [39], minimum tree principle [40], tabu search (TS), Pareto ant colony algorithm (HPACA) [41], and the GA-DBPSO algorithm [42,43]. Changing the airflow in any one of the tunnels leads to changes in other associated tunnels. The sensitivity is used for measuring the airflow relationship between all the tunnels [44].

However, a mine ventilation system is a complex network, with different complexity levels in different areas. There are some problems with considering the accuracy to determine the minimum sensors’ location and reconstruct the air volume of all tunnels in the mines. In this study, we aimed to solve the problems of sensor optimization and air flow reconstruction, and realize the air volume monitoring of the whole air network economically and effectively. In this paper, we propose an algorithm for the well-posed reconstruction of the airflow in a mine ventilation network. The algorithm is based on the structure of the underlying graph, and makes it possible to have a unique, optimized solution to the air volume of tunnels. Compared with other methods, this algorithm involves lower costs and a simpler process.

The rest of this paper is organized as follows. We state the problems we aimed to study, provide a theoretical analysis of our research, and explain some concepts that need to be used in Section 2. In Section 3, we introduce the methods of sensor location optimization and flow reconstruction. The above-mentioned methods are transformed into algorithms, and the specific calculation process is expressed by matrices and formulas, which are verified by taking a single-source and single-sink network as an example in Section 4 and Section 5. In Section 6, we verify the algorithm by taking multisource and multisink network by the ventilation data of a coal mine. The main conclusions of this paper are given in Section 7.

## 2. Problem Statement

Our goal was to locate wind speed sensors so that the airflow of a ventilation network could be inferred from the measurements while minimizing the number of sensors used. We propose a well-posed flow reconstruction algorithm, which uses the flow conservation equations and breadth-first search algorithm to search the whole ventilation network, establish the equations, and then solve the airflow reconstruction problem.

### 2.1. Possibility Analysis on Well-Posed Flow Reconstruction

**Figure 2:**
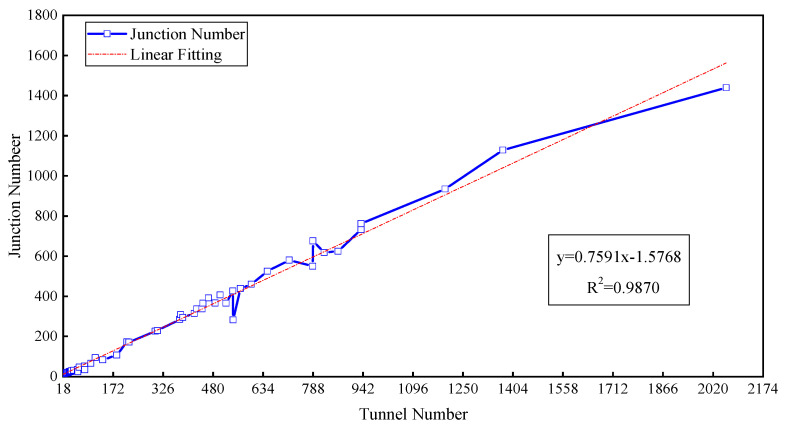
Correlation between the junction number and tunnel number.

**Figure 3:**
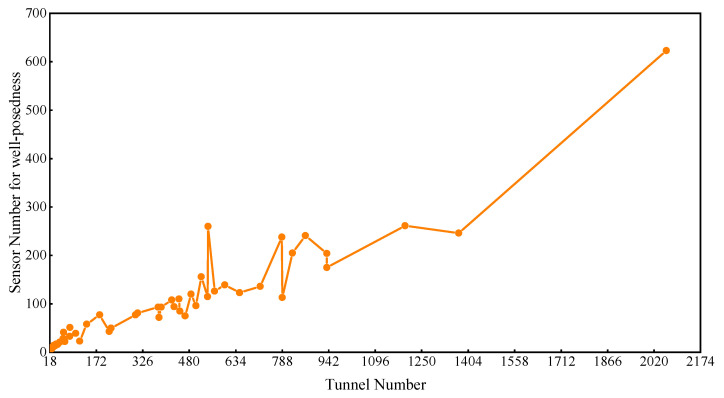
The number of sensors required for well-posed reconstruction.

The correlation between the junction number and tunnel number was obtained, as shown in Figure 2. The junction number increases linearly with the tunnel number. This means that there is more mass conservation at the junctions as the tunnel number increases. For a mine ventilation network with a single sink and a single source, *m* − 1 mass conservation equations can be established for *m* junctions with a connecting sink and source as a virtual junction. There are *n* tunnels in the mine, and *n* unknown parameters, so *n* − *m* + 1 wind speed sensors are needed for the well-posed flow reconstruction if the sensors are set up accurately. Similarly, *n* − *m* + *k* − 1 wind speed sensors are used for multisinks and a multisource network (*k* denotes the total number of the sinks and sources). The above analysis indicates that the minimum number of sensors needed for a well-posed solution may be under 100 when the tunnel number is less than 380, while more than 300 sensors need to be installed when the tunnel number is over 1500; 100–300 sensors are needed if the range of tunnel numbers is 380–1500, as shown in Figure 3. This shows that the calculation complexity is also affected by the network structure.

**Figure 4:**
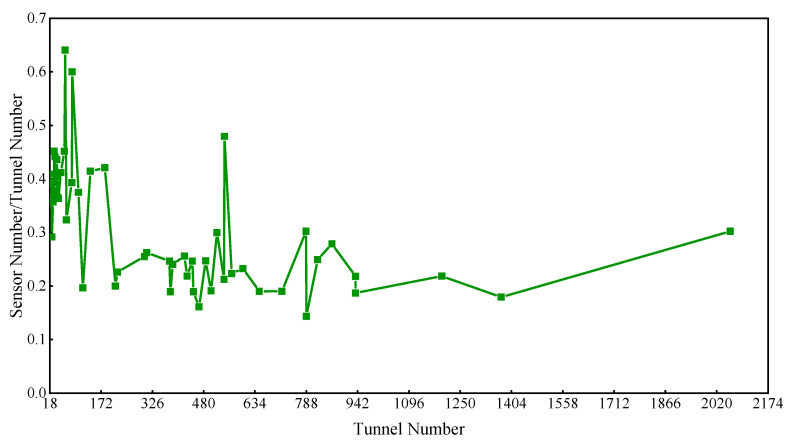
Ratio of sensor to tunnel number.

Figure 4 shows that the ratio of sensor to tunnel number (RST) for well-posed reconstruction is over 30% and even up to 63% when there are fewer than 150 tunnels in the mine, while the RST is below 30% when the tunnels number more than 150. The above data demonstrated that sensors in some mines can be set up for well-posed flow reconstruction where the RST is less than 30% or there are few tunnels. However, there will be no solution or multiple solutions for the reconstruction if the locations of sensors are inaccurate. Therefore, the installation locations of sensors are critical for flow reconstruction.

### 2.2. Flow Conservation Equation

Ventilation networks are represented by means of a directed graph:

(1) G=(V,E),

where

$V=\left\{v_{1},v_{2},\cdots ,v_{m}\right\}$ corresponds to the set of junctions,

m is the junction number,

$E=\left\{e_{1},e_{2},\cdots ,e_{n}\right\}$ represents the set of tunnels, and

n is the tunnel number. 

The out branches set is denoted as $E^{+}\left(v_{i}\right),\left(v_{i},v_{j}\right)\in E^{+}\left(v_{i}\right)$. The in branches set is denoted as $E^{-}\left(v_{i}\right),\;\left(v_{k},v_{i}\right)\in E^{-}\left(v_{i}\right)$. {s}, {t} are the set of the source junctions and the sink junctions, respectively. For any junction v∉{s,t}, inflow equals outflow:

(2) $$\sum \rho_{ij}q_{ij}=\sum \rho_{ki}q_{ki}.$$

A generalized equation of mass can be derived from Equation (2) and the algebraic sum of the tunnels’ flow mass is $Q_{t}\;$in any directed cut set of the network with sinks and sources:

(3) $$SQ^{T}={\left(\sum_{j=1}^{n}s_{ij}q_{j}\right)}_{s\times 1}=Q_{t},$$

where 

$S={\left(s_{ij}\right)}_{s\times n}$ represents the directed cut set;

s is the cut set number;

$Q_{t}$ is the total mass of the network. 

### 2.3. Improved Breadth-First Search

An exact mathematical model (i.e., a direct solution) may not be found for some problems. In this case, search methods are generally used to solve the issues, among which a breadth-first search (BFS) is the simplest method.

A BFS is one of the graph algorithms. The process is to search for every possible edge by traditional BFS; each junction can only be visited once. The basic idea is to start from a junction *v* in the graph. In turn, the graph is traversed in breadth first from the unreachable adjacent junctions of *v* until the junctions in the graph are connected with the paths of *v* visited. If any junctions in the graph are not visited at this time, the breadth-first traversal is performed again from a junction that has not been visited until all the junctions in the graph have been accessed.

**Figure 5:**
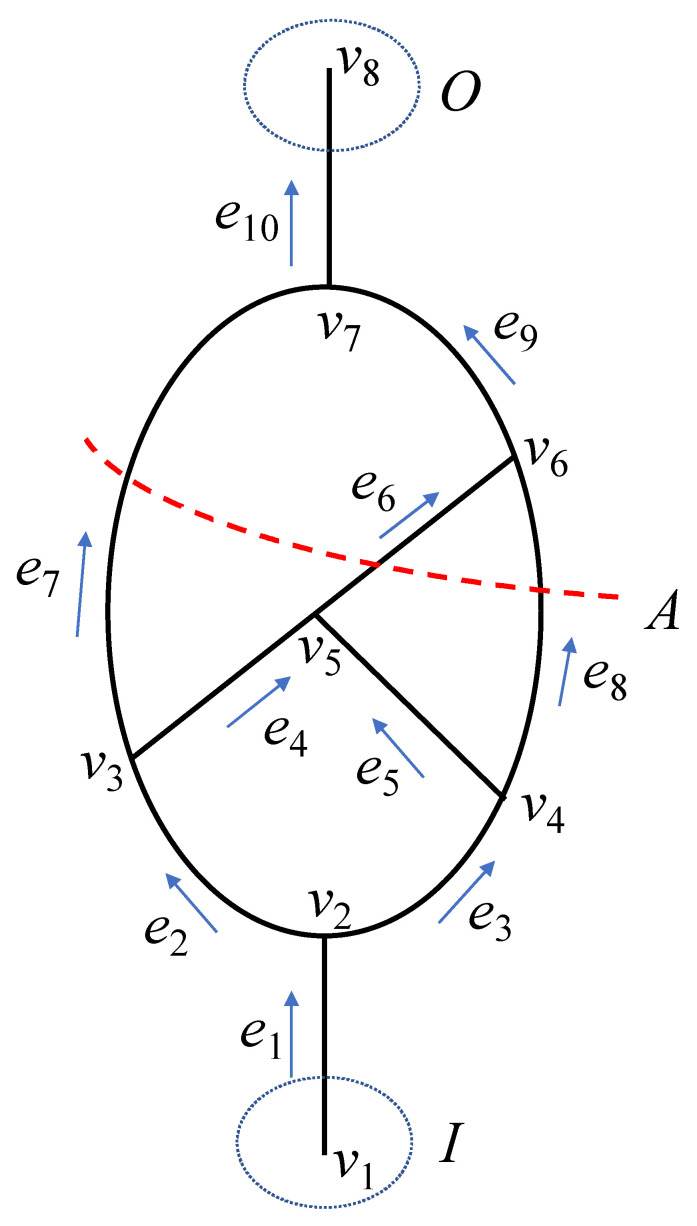
Schematic diagram of independent cut set. (Red line A represents cut line, and I and O represent source junction and sink junction respectively).

Figure 5 is a ventilation network diagram of single source and single sink. It shows a schematic diagram of an independent cut set. If we launch a BFS from junction $v_{2}$ (the following access order is not unique, and the second point can be either $v_{3}$ or $v_{4}$), we may get an access process as follows: $v_{2}-v_{3}-v_{7}-v_{8}$. We then go back to $v_{3}$. We can get the access process as follows: $v_{2}-v_{3}-v_{5}-v_{6}-v_{7}-v_{8}$. For the same reason, we can go back to $v_{2}$. We can then continue to search $v_{2}-v_{4}-v_{6}-v_{7}-v_{8}$. The search is finished when all junctions are visited. The result of each BFS must be a connected component of the graph. 

According to the analysis in Section 2.1, the spanning tree structure meets the requirements of well-posed reconstruction. Therefore, an improved breadth-first search (IBFS) method is proposed, based on the spanning tree structure instead of the whole network. 

An IBFS is a search algorithm based on the spanning tree structure. The first step of an IBFS is to determine the spanning tree and sort the junctions and tunnels of the spanning tree. Then, all source and sink junctions of the spanning tree must be determined. The source junction is taken as the starting junction to start the search, and the incidence branches of the junction are searched. After searching all incidence branches, the search must be continued according to the previous step with the end junction of the incidence branch as the starting junction until the sink junction is searched. The previous junction must then be considered as the starting junction, and other branches are searched for. If there are no other branches, the retreat is continued until the starting junction with other branches is found and the search is maintained. When all branches of the spanning tree are searched, the search ends.

Take Figure 5 as an example; $e_{1},e_{2},e_{3},e_{4},e_{6},e_{9},e_{10}$ form a spanning tree. If we launch an IBFS from junction $v_{1}$, we may get an access process as follows: $v_{1}-v_{2}-v_{3}-v_{4}$. Because $v_{4}$ is a sink of the spanning tree, we need to go back to $v_{2}$. The adjacent junction $v_{3}$ of junction $v_{2}$ has an incidence branch. The next search starts from $v_{3}$. The process is as follows: $v_{3}-v_{5}-v_{6}-v_{7}-v_{8}$. When all branches of the spanning tree have been searched, the search ends. The IBFS is the basis of our research on sensor optimization and air volume reconstruction. The following algorithms need to use IBFS.

### 2.4. Definition of Single Junction Cut Sets

In the directed graph $G=\left(V,E\right),\;\;v_{i}\in v,$ after deleting junction $v_{i}$, all tunnels associated with $v_{i}$ from the graph cannot be connected. This is called a single junction cut set ($S=D_{v_{i}}$).

In Figure 5, $V=\left\{v_{1},v_{2},v_{3},v_{4},v_{5},v_{6},v_{7},v_{8}\right\}\;\mathrm{and}\;m=8$. In other words, there are eight single junction cut sets in the directed graph:

G=(V,E),

$D=\left\{D_{v_{1}},D_{v_{2},}D_{v_{3}},D_{v_{4}},D_{v_{5}},D_{v_{6}},D_{v_{7}},D_{v_{8}}\right\}$,

$D_{v_{1}}=\left\{e_{1}\right\}$,

$D_{v_{2}}=\left\{e_{1},e_{2},e_{3}\right\}$,

$D_{v_{3}}=\left\{e_{2},e_{4},e_{7}\right\}$,

$D_{v_{4}}=\left\{e_{3},e_{5},e_{8}\right\}$,

$D_{v_{5}}=\left\{e_{4},e_{5,}e_{6}\right\}$,

$D_{v_{6}}=\left\{e_{6},e_{8},e_{9}\right\}$,

$D_{v_{7}}=\left\{e_{7},e_{9},e_{10}\right\}$,

$D_{v_{8}}=\left\{e_{10}\right\}$.

### 2.5. Definition of Independent Cut Set

A new concept called independent cut set is proposed. The source–sink matrix describes the ventilation flow in the mine. The source junction set is *I*, and the sink junction set is *O.* In the mine ventilation network G=(V,E), I⊂V, O⊂V, a cut C=(S,T) partitions V into two subsets, S and T. The cut set A of a cut C=(S,T) is the set {(u,v)ϵE|uϵS, vϵT, I⊂S,O⊂T}, which is called the independent cut set. The independent cut set can divide the input and output junctions into two disjointed parts, which means that the removal of all edges disconnects all paths from the input to output junctions, as shown in Figure 5. 

### 2.6. Algorithm of Independent Cut Set

An independent cut set search algorithm for a single-source, single-sink ventilation network was studied. The graph is described as:

G=(V,E),

$E=\left\{e_{1},e_{2},\ldots \ldots ,e_{n}\right\}$,

n=|E|,

*V*$=\left\{v_{1},v_{2},\ldots \ldots ,v_{m}\right\},$ and m=|V|. 

**Figure 6:**
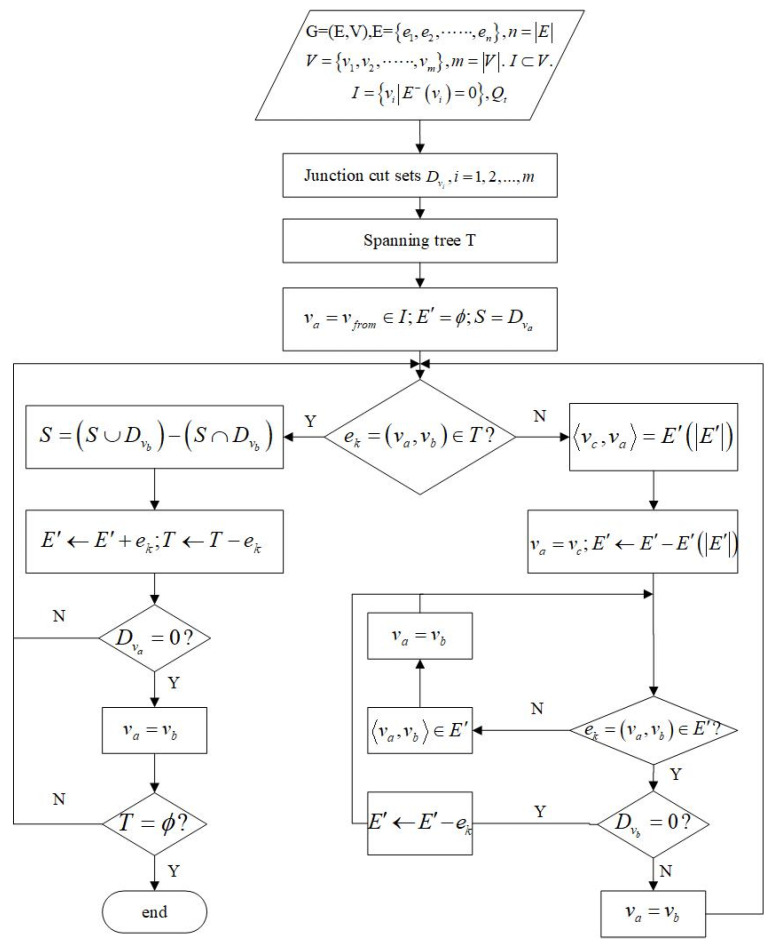
Flow diagram for the independent cut set.

The sources set is denoted by $I\subset V,\;I=\left\{v_{i}|E^{-}\left(v_{i}\right)=0\right\},$ where O represents the sink set, $O=\left\{v_{i}|E^{+}\left(v_{i}\right)=0\right\}\subset V$. First, single-junction cut sets $D_{v_{2}},\;i=1,2,\ldots ,m$, were obtained, such as $D_{v_{2}}=\left\{e_{1},e_{2},e_{3}\right\}$ for the junction $v_{2}$. A spanning tree that connects all junctions in the mine network without forming a cycle was created, including some tunnels on demand. The spanning tree was then changed to an undirected graph. The breadth-first search starts from the source junction $S=D_{v_{1}}$. When it comes to junction $v_{b}$ in the spanning tree, the independent cut set is calculated by $S=\left(S\cup D_{v_{b}}\right)-\left(S\cap D_{v_{b}}\right)$. It was followed until the searching of all the junctions of the spanning tree was completed (see Figure 6).

Take the network shown in Figure 5, for example; the network graph is:

G=(V,E),

$E=\left\{e_{1},e_{2},e_{3},\;e_{4},e_{5},e_{6},e_{7},e_{8},e_{9},e_{10}\right\},n=10,$

$V=\left\{v_{1},v_{2},v_{3},v_{4},v_{5},v_{6},v_{7},v_{8}\right\},m=8,$

$I=\left\{e_{1}\right\}$,

$O=\left\{e_{10}\right\}$.

The spanning tree is:

$\;T=\{e_{1},e_{2},e_{3},e_{4},e_{6},e_{9},e_{10}\}$.

The single junction cut sets for every junction are:

$D_{v1}=\left\{e_{1}\right\},$

$D_{v2}=\left\{e_{1},e_{2},e_{3}\right\},$

$D_{v3}=\left\{e_{2},e_{4},e_{7}\right\},$

$D_{v4}=\left\{e_{3},e_{5},e_{8}\right\},$

$D_{v5}=\left\{e_{4},e_{5},e_{6}\right\},$

$D_{v6}=\left\{e_{6},e_{8},e_{9}\right\},$

$D_{v7}=\left\{e_{7},e_{9},e_{10}\right\},$

$D_{v8\;}=\left\{e_{10}\right\}$.

According to the flow diagram, the process of obtaining the independent cut set is as shown in Table 1.

## 3. Methods of Location Optimization and Flow Reconstruction

### 3.1. Location Optimization of Wind Speed Sensors

In order to study the minimum number and location of sensors that can completely recover the airflow in a mine network, an algorithm based on the topological structure of the network and the information was developed. The algorithm creates spanning trees and uses the IBFS, a well-known graph traversal algorithm, to search. The inputs of the algorithm on the location of wind speed sensors are the directed graph G=(V,E) and single junction cut set, while the output is the independent cut set. The procedures of the algorithm are as follows:

1. Change all junctions in V with a single junction and find the single-junction cut set of all junctions.
2. Initialize $V_{a}=V_{from}$.
3. Use IBFS over spanning tree starting at $v_{a}$. Denote $e_{k}$ and $v_{b}$ as the visited edges and junctions, respectively.
4. Obtain the independent cut set S while $e_{k}\epsilon T$: end if Tϵθ; otherwise, search the next spanning tree. To avoid repeated searches, the edges and junctions passing by should be recorded for every independent cut set obtained.
5. If any junctions in the graph are not visited at this time, the breadth-first traversal must be performed again from a junction that has not been visited until all the junctions in the graph have been accessed.
6. Put the known air volume into Equation (3) to solve for the unknown air volume.

### 3.2. Flow Reconstruction Method by the Wind Speed Sensors

According to the Chinese industry standards GB/T51272-2018 and AQ2031-2011, it is compulsory to install wind speed sensors in main return tunnels, the return tunnels of all mining areas, and the return tunnels of all sublevels. These tunnels are seen as cotrees for the spanning tree, which can be generated according to the cotrees by the Kruskal algorithm.

As shown in Figure 6, the independent cut sets can be searched by the spanning tree. There is a flow equation, Equation (3), for every independent cut set. Taking Table 1 as an example, seven flow equations can be set. $Q_{t}$ is the air volume of the main fan that must be monitored. It is assumed that $Q_{t}=15,\;Q_{5}=4$, $Q_{7}=5$, and $Q_{8}=2$.

(4) $$\{\begin{array}{l} \;Q_{1}=Q_{t}\\ \;Q_{2}+Q_{3}=Q_{t}\\ \;Q_{3}+Q_{4}+Q_{7}=Q_{t}\\ \;Q_{4}+Q_{7}+Q_{5}+Q_{8}=Q_{t}\\ \;Q_{6}+Q_{7}+Q_{8}=Q_{t}\\ \;Q_{7}+Q_{9}=Q_{t}\\ \;Q_{10}=Q_{t} \end{array}$$

Equation (4) is solved by the Gaussian elimination method. The air volume of the tunnels in the spanning tree is $Q_{1}=15$, $Q_{2}=9$, $Q_{3}=6$, $Q_{4}=4$, $Q_{6}=8$, $Q_{9}=10$, and $Q_{10}=15$.

## 4. Algorithm Optimization of Sensor Location Problem

In this paper, we propose an independent cut set algorithm for well-posed reconstruction of the airflow in a mine ventilation network; however, its process is too complex. For a large-scale ventilation network, the calculation amount and complexity are very large. In order to simplify the calculation process, the independent cut set algorithm can be optimized by matrixing the calculation process, i.e., all equations in the calculation process can be expressed by matrix. With the help of matrix characteristics, the sensor location problem can be calculated more quickly and intuitively. In addition, there is a mature calculation program for the matrix problem. It is more convenient to solve the matrix problem by computer than to directly solve the newly defined algorithm. Especially for large-scale network problems, faster and more accurate calculations can be made.

### 4.1. Optimization of Single Junction Cut Set

In order to more clearly represent the single junction cut sets, we can use the complete incidence matrix to express the single junction cut sets of each junction. The complete incidence matrix is a 0-1-(−1) matrix that describes the mine ventilation network structure through the spatial relationships between the tunnels and junctions of the network. Each column element of the matrix represents each tunnel of the mine ventilation network. Each row element denotes the junction. The matrix can be expressed as follows:

(5) $$L_{m\times n}=\left(\begin{array}{ccc} \begin{array}{c} l_{11}\\ \vdots \end{array} & \begin{array}{cc} \ldots & l_{1k}\\ \ddots & \vdots \end{array} & \begin{array}{cc} \ldots & l_{1n}\\ ⋰ & \vdots \end{array}\\ l_{j1} & \begin{array}{cc} \cdots & l_{jk} \end{array} & \begin{array}{cc} \cdots & l_{jn} \end{array}\\ \begin{array}{c} \vdots \\ l_{m1} \end{array} & \begin{array}{cc} ⋰ & \vdots \\ \cdots & l_{mk} \end{array} & \begin{array}{cc} \ddots & \vdots \\ \cdots & l_{mn} \end{array} \end{array}\right),$$

where $L_{m\times n}$ is the complete incidence matrix of the network of dimension (m×n) and $l_{ij}$ is 0, 1, or −1. $l_{ij}=1$ indicates that $e_{j}$ and junction $v_{i}$ are associated, and $e_{j}$ is the out branch of $v_{i}$. $l_{ij}=-1$ indicates that $e_{j}$ is the in branch of $v_{i}$. Through the definition of single junction cut sets, if branch $e_{j}$ and junction $v_{i}$ are associated, then $e_{j}$ belongs to $D_{v_{i}}$. Therefore, $e_{j}$ is an element of $D_{v_{i}}$ when $\left|l_{ij}\right|=1$. On the contrary, $l_{ij}=0$ indicates that $e_{j}$ and junction $v_{i}$ are not related.

The matrix can be represented through a set of column vectors. $L_{m\times n}$ denotes the complete incidence matrix with m junctions and n tunnels, and can be expressed as follows:

(6) $$L_{m\times n}={\left[L_{1}L_{2}\cdot \cdot \cdot L_{j}\cdot \cdot \cdot L_{m}\right]}^{T},$$

where $L_{j}$ is the $j_{th}$ row vector of dimension (1×n). $D_{v_{j}}=\left\{e_{k}|e_{k}=E\left(v_{j}\right)\right\}$, i.e., $D_{v_{j}}=\left\{e_{k}|l_{jk}\neq 0\right\}$ (j=1,2,⋯,m; k=1,2,⋯,n). $e_{k}$, corresponding to all nonzero entries $l_{jk}$ in $L_{j}$, is the single-junction cut set corresponding to junction $v_{j}$; that is, $e_{k}$ is all the elements of $D_{v_{j}}$.

### 4.2. Optimization of Independent Cut Set Algorithm

In Section 2.6, it is revealed that the independent cut set is calculated by $S=\left(S\cup D_{v_{b}}\right)-\left(S\cap D_{v_{b}}\right)$, which is a equation related to the single junction cut set and independent cut set. From Section 4.1, we know the matrix form of the single junction cut sets $D_{v_{j}}$, i.e., complete incidence matrix (see Equations (5) and (6)). Therefore, we can convert the solution process of the independent cut set into matrix form. An independent cut set–branch incidence matrix can be established to obtain the independent cut set of the ventilation network. The matrix can be expressed as follows:

(7) $$S_{m\times n}=\left(\begin{array}{ccc} \begin{array}{c} s_{11}\\ \vdots \end{array} & \begin{array}{cc} \ldots & s_{1k}\\ \ddots & \vdots \end{array} & \begin{array}{cc} \ldots & s_{1n}\\ ⋰ & \vdots \end{array}\\ s_{j1} & \begin{array}{cc} \cdots & s_{jk} \end{array} & \begin{array}{cc} \cdots & s_{jn} \end{array}\\ \begin{array}{c} \vdots \\ s_{m1} \end{array} & \begin{array}{cc} ⋰ & \vdots \\ \cdots & s_{mk} \end{array} & \begin{array}{cc} \ddots & \vdots \\ \cdots & s_{mn} \end{array} \end{array}\right),$$

where $S_{m\times n}$ is the independent cut set–branch incidence matrix of the network of dimension (m×n) and $s_{ij}$ is 0 or 1. $s_{ij}=1$ indicates that $e_{j}$ is a subset of the independent cut set $S_{i}$ at junction $v_{i}$. $s_{ij}=0$ denotes that $e_{j}$ does not belong to the independent cut set $S_{i}$ at junction $v_{i}$. Compared with the complete incidence matrix, each column element of the new matrix still represents each branch of the ventilation network. However, row elements are expressed by independent cut sets S. In Section 4.1, the complete incidence matrix (see Equation (6)) is expressed as follows:

$$L_{m\times n}={\left[L_{1}L_{2}\cdot \cdot \cdot L_{j}\cdot \cdot \cdot L_{m}\right]}^{T}.$$

Through the calculation formula of independent cut sets $S=\left(S\cup D_{v_{b}}\right)-\left(S\cap D_{v_{b}}\right)$, we can obtain the matrix expression:

(8) $$\{\begin{array}{l} S_{j+1}=S_{j}+L_{j+1}\\ S_{1}=L_{1} \end{array}\left(j=1,2,\cdots ,m-1\right),$$

where $e_{k}$ corresponds to all nonzero entries and $s_{jk}$ in $S_{j}$ is the independent cut sets. According to Equation (8), we can obtain the matrix form of the independent cut set.

## 5. Sensor Location and Flow Reconstruction Based on Algorithm Optimization

### 5.1. Sensor Location Based on Algorithm Optimization

Take the network shown in Figure 5 as an example; according to Equation (1), the network graph is:

$$\begin{array}{c} G=\left(V,E\right);\\ E=\left\{e_{1},e_{2},e_{3},\;e_{4},e_{5},e_{6},e_{7},e_{8},e_{9},e_{10}\right\},n=10;\\ V=\left\{v_{1},v_{2},v_{3},v_{4},v_{5},v_{6},v_{7},v_{8}\right\},m=8. \end{array}$$

The junctions and tunnels have been sorted in Figure 5. The whole calculation process is carried out according to the IBFS. According to Equations (5) and (6), we can obtain the single junction cut set expressed by the matrix, that is, the complete incidence matrix of ventilation network G=(V,E). In order to facilitate the presentation of the row vector and column vector corresponding to each element of the complete incidence matrix, we show the complete incidence matrix corresponding to Figure 5 in the form of Table 2.

The matrix can be expressed as $L_{8\times 10}$. According to Equation (6), the form of its column vector is $L_{8\times 10}={\left[L_{1}\;L_{2}\;\cdot \cdot \cdot \;L_{j}\;\cdot \cdot \cdot \;L_{8}\right]}^{T}$. All nonzero entries in $L_{1}$ are the result of $D_{v_{1}}$, that is, $L_{1}=\left\{1\;0\;0\;0\;0\;0\;0\;0\;0\;0\right\}$; the first single junction cut set is $D_{v_{1}}=\left\{e_{1}\right\}.$ Similarly, all nonzero entries in $L_{2}$ are the result of $D_{v_{2}}$, that is, $L_{2}=\left\{-1\;1\;1\;0\;0\;0\;0\;0\;0\;0\right\},$ and the second single junction cut set is $D_{v_{2}}=\left\{e_{1},e_{2},e_{3}\right\}$, etc. Through the matrix, we can easily obtain the single-junction cut sets of each junction, and the result conforms to the definition of single-junction cut sets.

In order to obtain the independent cut set, $L_{8\times 10}$ is substituted into Equation (8). The results are shown in Table 3.

The matrix can be expressed as $S_{8\times 10}$. The form of its column vector is $S_{8\times 10}={\left[s_{1}\;s_{2}\;\cdot \cdot \cdot \;s_{j}\;\cdot \cdot \cdot \;s_{8}\right]}^{T}$. All nonzero entries in $s_{j}$ are the results of the independent cut set. For example, $s_{1}=\left\{1\;0\;0\;0\;0\;0\;0\;0\;0\;0\right\}$ and the first independent cut set is $S_{1}=\left\{e_{1}\right\}$; $s_{2}=\left\{0\;1\;1\;0\;0\;0\;0\;0\;0\;0\right\}$ and the second independent cut set is $S_{2}=\left\{e_{2},\;e_{3}\right\}$; $s_{3}=\left\{0\;0\;1\;1\;0\;0\;1\;0\;0\;0\right\}\;$and the first independent cut set is $S_{1}=\left\{e_{3},e_{4},\;e_{7}\right\}$, etc.

Through comparison, it can be seen that the results obtained through the matrix in Table 3 are consistent with those in Table 1.

### 5.2. Flow Reconstruction Based on Algorithm Optimization

In order to simplify the calculation process, the solution of the independent cut set is obtained by a matrix. Therefore, we can also express the calculation of Equation (3) by a matrix. According to Equation (3), the air volume in each tunnel can be denoted by air volume set Q, and its expression is as follows:

(9) $$Q^{T}=\left\{Q_{1}\;Q_{2}\;\cdots \;Q_{n}\right\},$$

where n denotes the tunnel number and $Q_{n}$ represents the air volume of the nth tunnel. According to Equation (3), the following equation can be obtained:

(10) $$SQ=Q_{t_{m\times 1}},$$

where $Q_{t_{m\times 1}}$ is the column vector of dimension (m×1), $Q_{t_{m\times 1}}={\left\{Q_{t}\;Q_{t}\;\cdots \;Q_{t}\right\}}^{T}$

.

In Figure 5, $S=S_{8\times 10}={\left[s_{1}s_{2}\;\cdot \cdot \cdot \;s_{8}\right]}^{T}$ and $Q={\left\{Q_{1}\;Q_{2}\;\cdots \;Q_{8}\right\}}^{T}$. It is assumed that $Q_{t}=15,\;Q_{5}=4$, $Q_{7}=5$, and $Q_{8}=2$. From Equation (10),

$$\left\{\begin{array}{ccc} \begin{array}{ccc} 1 & 0 & 0\\ 0 & 1 & 1 \end{array} & \begin{array}{ccc} 0 & 0 & 0\\ 0 & 0 & 0 \end{array} & \begin{array}{ccc} 0 & 0 & \begin{array}{cc} 0 & 0 \end{array}\\ 0 & 0 & \begin{array}{cc} 0 & 0 \end{array} \end{array}\\ \begin{array}{ccc} 0 & 0 & 1\\ 0 & 0 & 0\\ 0 & 0 & 0 \end{array} & \begin{array}{ccc} 1 & 0 & 0\\ 1 & 1 & 0\\ 0 & 0 & 1 \end{array} & \begin{array}{ccc} 1 & 0 & \begin{array}{cc} 0 & 0 \end{array}\\ 1 & 1 & \begin{array}{cc} 0 & 0 \end{array}\\ 1 & 1 & \begin{array}{cc} 0 & 0 \end{array} \end{array}\\ \begin{array}{ccc} 0 & 0 & 0\\ 0 & 0 & 0\\ 0 & 0 & 0 \end{array} & \begin{array}{ccc} 0 & 0 & 0\\ 0 & 0 & 0\\ 0 & 0 & 0 \end{array} & \begin{array}{ccc} 1 & 0 & \begin{array}{cc} 1 & 0 \end{array}\\ 0 & 0 & \begin{array}{cc} 0 & 1 \end{array}\\ 0 & 0 & \begin{array}{cc} 0 & 0 \end{array} \end{array} \end{array}\right\}\left\{\begin{array}{c} Q_{1}\\ Q_{2}\\ \begin{array}{c} Q_{3}\\ Q_{4}\\ \begin{array}{c} Q_{5}\\ Q_{6}\\ \begin{array}{c} Q_{7}\\ Q_{8} \end{array} \end{array} \end{array} \end{array}\right\}=\left\{\begin{array}{c} Q_{t}\\ Q_{t}\\ \begin{array}{c} Q_{t}\\ Q_{t}\\ \begin{array}{c} Q_{t}\\ Q_{t}\\ \begin{array}{c} Q_{t}\\ Q_{t} \end{array} \end{array} \end{array} \end{array}\right\}$$

After simplifying the above formula, the following equation can be obtained:

$$\begin{array}{l} \;Q_{1}=Q_{t}\\ \;Q_{2}+Q_{3}=Q_{t}\\ \;Q_{3}+Q_{4}+Q_{7}=Q_{t}\\ \;Q_{4}+Q_{7}+Q_{5}+Q_{8\;}=Q_{t}\\ \;Q_{6}+Q_{7}+Q_{8}=Q_{t}\\ \;Q_{7}+Q_{9}=Q_{t}\\ \;Q_{10}=Q_{t} \end{array}.$$

The air volume of the tunnels in the spanning tree is $Q_{1}=15$, $Q_{2}=9$, $Q_{3}=6$, $Q_{4}=4$, $Q_{6}=8$, $Q_{9}=10$, and $Q_{10}=15$.

## 6. Case Study

**Figure 7:**
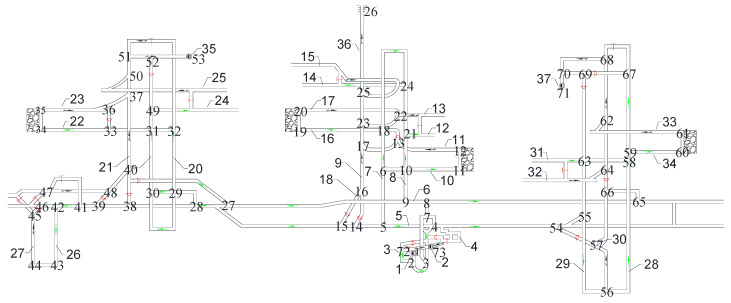
A coal mine ventilation system used by Jinmei Co., Ltd. (Red indicates the position of air doors or wind windows, and black arrows indicate the exhaust air flow. Green is the incoming air flow as well as the fresh air flow. Each number indicates the junction number and tunnel number).

**Figure 8:**
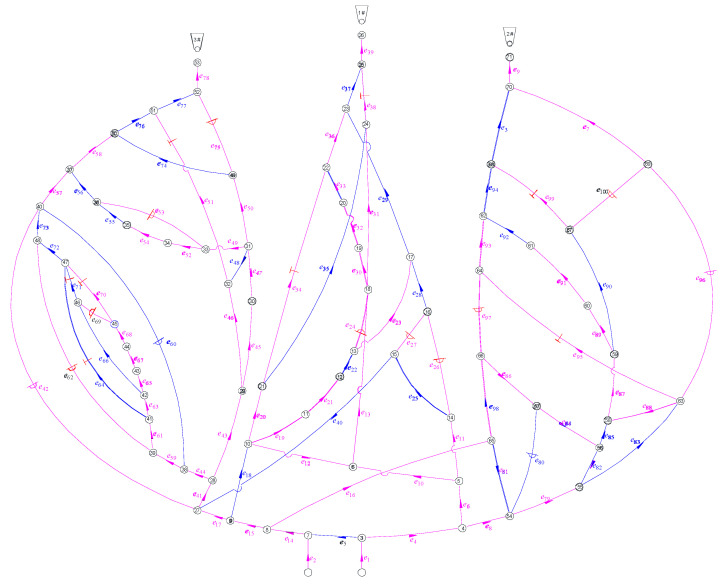
Coal mine ventilation used by Jinmei Co., Ltd. The magenta represents spanning trees and the blue represents cotrees.

According to the standards AQ1028-2006 and GB/T 10178, the ventilation network is a fluid network formed by conveying air flow pipelines; various regulating facilities, power facilities, and air flow pipelines are connected. Figure 7 is the ventilation network of a coal mine run by Jinmei Co., Ltd. G=(V,E), where the tunnel number is |E|=100, and the junction number is |V|=71. Table 4 shows the whole tunnel’s reconstructed air volume and simulated air volume. The test air volume [45] is obtained by a mine ventilation simulation system (MVSS) [46]. The principle of solving the network by MVSS is to establish the mathematical equations for the ventilation resistance law, the air volume balance law, the air pressure balance law, the known total air volume of the fans, and the ventilation resistance coefficient, and then solve them. The main fans are installed in $e_{9}$, $e_{39}$, and $e_{78}$, and their characteristic equations are shown in Equation (11). For a multisource and multisink ventilation network, all sources and sinks should be connected to become a single-source and single-sink network. In a ventilation network, if the spanning tree is removed, the remaining part is called the cotree. As shown in Figure 8, 33 wind speed sensors are used for the network, including 30 cotrees and 3 wind shafts with fans. The tunnels that must be set up with wind speed sensors are$\;e_{3}$, $e_{9}$, $e_{22}$, $e_{28}$, $e_{33}$, $e_{39}$, $e_{55}$, $e_{68}$, $e_{72}$, $e_{76}$, $e_{78}$, $e_{84}$, $e_{92}$, and $e_{94}$. A spanning tree is generated that does not include the above tunnels (the magenta in Figure 8). Considering the difficulty of testing the lower wind speed, most of the cotrees should not contain the tunnels with structures such as throttles and confined walls. Based on the flow diagram in Figure 5, the air volume of the spanning trees can be calculated by the linear flow equation system of the independent cut sets.

(11) $$\{\begin{array}{c} H\left(q_{9}\right)=1932.25+44.84q_{9}-0.64q_{9}^{2}\\ H\left(q_{39}\right)=1932.25+44.84q_{39}-0.64q_{39}^{2}\\ H\left(q_{78}\right)=1932.25+44.84q_{78}-0.64q_{78}^{2} \end{array}$$

The reconstructed air volume in Table 4 was obtained by the independent cut set algorithm. Similarly, the results obtained by the independent cut set algorithm followed the three basic laws of air volume distribution. Their basic principles are similar. We also used part of the test air volume as a known quantity for the calculation. Therefore, their calculation results should be the same; otherwise, they will prove that the independent cut set algorithm is incorrect. It can be seen from Table 4 that their results were exactly the same. It is feasible to use the independent cut set algorithm to determine the minimum number and location of sensors through the case study. By placing sensors on the cotrees, the air volume of the spanning trees can be obtained. Due to the uniqueness of the well-posed solution, the error of flow reconstruction is zero (see Table 4). There will be no errors or other solutions.

Figure 5 is an example of a simple network with a single source and single sink, and Figure 8 is an example of a complex ventilation network with multiple sources and multiple sinks. Through the calculation of these two examples, it was found that the independent cut-set algorithm was suitable for both of the examples. Therefore, the independent cut set algorithm can be used to solve the sensor optimization and air volume reconstruction problems of all types of ventilation networks. In line with previous research, the calculation complexity was also affected by the network structure. Due to the uniqueness of the well-posed solution, the error of the result can only come from the measurement accuracy of the sensor and will not be affected by other factors. Moreover, the existing solution is unique and has no errors.

## 7. Conclusions

In this research, the wind speed sensor location problem of the mine ventilation network was solved by using the independent cut set algorithm, which is a new concept based on the structure of the underlying graph. Firstly, we found the problems: the sensor number was n−m+k−1, and we located flow sensors in the cotrees that must be set up with sensors according to the Chinese standards. The calculation complexity was affected by the network structure. Secondly, we discussed the possibility of well-posed reconstruction based on the mine ventilation networks. Fewer than 30% of tunnels need to be set up with wind speed sensors to achieve a well-posed reconstruction of the airflow in all the tunnels if the mines have over 200 tunnels. For mines with fewer than 200 tunnels, more than 30% of tunnels should be installed with sensors. Due to the uniqueness of the well-posed solution, there is no error in the results unless the sensor accuracy does not meet the standard. Lastly, the algorithm of independent cut sets was presented for the well-posed reconstructions. The flow reconstructions were shown to be computationally efficient for the coal mine ventilation system used by Jinmei Co., Ltd. This algorithm works on velocity sensor distribution rather than sensors of hazardous and combustible gas. This algorithm is a well-posed reconstruction algorithm. It can only be used to determine the minimum number and location of sensors and cannot solve the underdetermined reconstruction of a given number of sensors. Therefore, the premise of using this algorithm is that the number of sensors will be sufficient. This study provides researchers with a sensor optimization scheme when the number of sensors is insufficient and provides the possibility for air volume reconstruction. At the same time, it reduces the cost for the air volume monitoring of the whole air network and provides some theoretical support for the realization of intelligent ventilation. In the following research, we will discuss the sensor location under the condition of underdetermined reconstruction.

## Figures and Tables

**Table 1:** The independent cut set of the graph in Figure 5.

No.	$v_{a}$	$e_{k}\in \langle v_{a},v_{b}\rangle \in T$	S	$E^{\prime }$	Search	$\left|D_{vb}\right|$
1			$S_{1}=D_{v1}=\left\{e_{1}\right\}$			0
2	$v_{1}$	$e_{1}\in \langle v_{1},v_{2}\rangle \in T$	$S_{2}=\left\{e_{2},e_{3}\right\}$	$e_{1}$	$v_{1}\;\overset{e_{1}}{\to }\;v_{2}$	0
3	$v_{2}$	$e_{2}\in \langle v_{2},v_{3}\rangle \in T$	$S_{3}=\left\{e_{3},e_{4},e_{7}\right\}$	$e_{1},e_{2}$	$v_{2}\;\overset{e_{2}}{\to }\;v_{3}$	1
4	$v_{2}$	$e_{3}\in \langle v_{2},v_{4}\rangle \in T$	$S_{4}=\left\{e_{4},e_{7},e_{5},e_{8}\right\}$	$e_{1},e_{2},e_{3}$	$v_{2}\;\overset{e_{3}}{\to }\;v_{4}$	0
5	$v_{3}$	$e_{4}\in \langle v_{3},v_{5}\rangle \in T$	$S_{5}=\left\{e_{7},e_{6},e_{8}\right\}$	$e_{1},e_{2},e_{3},e_{4}$	$v_{3}\;\overset{e_{4}}{\to }\;v_{5}$	0
6	$v_{5}$	$e_{6}\in \langle v_{5},v_{6}\rangle \in T$	$S_{6}=\left\{e_{7},e_{9}\right\}$	$e_{1},e_{2},e_{3},e_{4},e_{6}$	$v_{5}\;\overset{e_{6}}{\to }\;v_{6}$	0
7	$v_{6}$	$e_{9}\in \langle v_{6},v_{7}\rangle \in T$	$S_{7}=\left\{e_{10}\right\}$	$e_{1},e_{2},e_{3},e_{4},e_{6},e_{9}$	$v_{6}\;\overset{e_{9}}{\to }\;v_{7}$	0
8	$v_{7}$	$e_{10}\in \langle v_{7},v_{8}\rangle \in T$				0

**Table 2:** The complete incidence matrix (corresponding to Figure 5).

	Link	$e_{1}$	$e_{2}$	$e_{3}$	$e_{4}$	$e_{5}$	$e_{6}$	$e_{7}$	$e_{8}$	$e_{9}$	$e_{10}$
Junction											
$v_{1}$		1	0	0	0	0	0	0	0	0	0
$v_{2}$		−1	1	1	0	0	0	0	0	0	0
$v_{3}$		0	−1	0	1	0	0	1	0	0	0
$v_{4}$		0	0	−1	0	1	0	0	1	0	0
$v_{5}$		0	0	0	−1	−1	1	0	0	0	0
$v_{6}$		0	0	0	0	0	−1	0	−1	1	0
$v_{7}$		0	0	0	0	0	0	−1	0	−1	1
$v_{8}$		0	0	0	0	0	0	0	0	0	−1

**Table 3:** Independent cut set–branch incidence matrix (corresponding to Figure 5).

	$e_{j}$	$e_{1}$	$e_{2}$	$e_{3}$	$e_{4}$	$e_{5}$	$e_{6}$	$e_{7}$	$e_{8}$	$e_{9}$	$e_{10}$
$s_{i}$											
$s_{1}$		1	0	0	0	0	0	0	0	0	0
$s_{2}$		0	1	1	0	0	0	0	0	0	0
$s_{3}$		0	0	1	1	0	0	1	0	0	0
$s_{4}$		0	0	0	1	1	0	1	1	0	0
$s_{5}$		0	0	0	0	0	1	1	1	0	0
$s_{6}$		0	0	0	0	0	0	1	0	1	0
$s_{7}$		0	0	0	0	0	0	0	0	0	1
$s_{8}$		0	0	0	0	0	0	0	0	0	0

**Table 4:** Ventilation system of Jinmei coal mine.

Edge	Test (m^3^/s)	Reconstruction (m^3^/s)	Edge	Test (m^3^/s)	Reconstruction (m^3^/s)	Edge	Test (m^3^/s)	Reconstruction (m^3^/s)
$e_{1}$	285.85	285.85	$e_{2}$	188.83	188.83	$e_{3}$	49.42	49.42
$e_{4}$	181.97	181.97	$e_{5}$	103.88	103.88	$e_{6}$	157.3	157.3
$e_{7}$	15.69	15.69	$e_{8}$	24.67	24.67	$e_{9}$	65.11	65.11
$e_{10}$	61.53	61.53	$e_{11}$	95.77	95.77	$e_{12}$	7.25	7.25
$e_{13}$	68.78	68.78	$e_{14}$	292.71	292.71	$e_{15}$	252.27	252.27
$e_{16}$	40.44	40.44	$e_{17}$	117.50	117.50	$e_{18}$	134.77	134.77
$e_{19}$	56.09	56.09	$e_{20}$	71.42	71.42	$e_{21}$	56.09	56.09
$e_{22}$	56.09	56.09	$e_{23}$	57.69	57.69	$e_{24}$	1.59	1.59
$e_{25}$	90.89	90.89	$e_{26}$	4.89	4.89	$e_{27}$	4.78	4.78
$e_{28}$	9.67	9.67	$e_{29}$	67.35	67.35	$e_{30}$	71.03	71.03
$e_{31}$	−3.84	−3.84	$e_{32}$	71.03	71.03	$e_{33}$	71.03	71.03
$e_{34}$	31.78	31.78	$e_{35}$	39.64	39.64	$e_{36}$	102.80	102.80
$e_{37}$	170.15	170.15	$e_{38}$	35.80	35.80	$e_{39}$	205.96	205.96
$e_{40}$	86.11	86.11	$e_{41}$	200.46	200.46	$e_{42}$	3.14	3.14
$e_{43}$	154.60	154.60	$e_{44}$	45.87	45.87	$e_{45}$	72.78	72.78
$e_{46}$	81.82	81.82	$e_{47}$	72.78	72.78	$e_{48}$	−1.89	−1.89
$e_{49}$	18.69	18.69	$e_{50}$	52.20	52.20	$e_{51}$	83.71	83.71
$e_{52}$	17.29	17.29	$e_{53}$	1.41	1.41	$e_{54}$	17.29	17.29
$e_{55}$	17.29	17.29	$e_{56}$	18.69	18.69	$e_{57}$	49.01	49.01

## Data Availability

The datasets generated and analyzed during the current study are available from the corresponding author upon reasonable request.
